# Supplementation of graded levels of rumen-protected choline to a pelleted total mixed ration did not improve the growth and slaughter performance of fattening lambs

**DOI:** 10.3389/fvets.2022.1034895

**Published:** 2022-11-23

**Authors:** Qin Huo, Xuezhao Sun, Tingting Wu, Zelin Li, Arjan Jonker, Peihua You, Rongquan Li, Jianping Li, Wannian Tian, Changsheng Li, Chunqing Wang, Yuhua He, Innocent Rugoho, Long Cheng, Meng You

**Affiliations:** ^1^The Innovation Center of Ruminant Precision Nutrition and Smart Farming, College of Animal Science and Technology, Jilin Agricultural Science and Technology University, Jilin, China; ^2^Jilin Inter-regional Cooperation Center for the Scientific and Technological Innovation of Ruminant Precision Nutrition and Smart and Ecological Farming, Jilin, China; ^3^AgResearch Limited, Grasslands Research Center, Palmerston North, New Zealand; ^4^Faculty of Veterinary and Agricultural Sciences, The University of Melbourne, Melbourne, VA, Australia; ^5^Portal Agri-Industries Co., Ltd., Nanjing, Jiangsu, China; ^6^Lely Australia Pty Ltd., Melbourne, VA, Australia

**Keywords:** rumen-protected choline, pelleted total mixed ration, growth performance, digestibility, growing lambs, meat quality

## Abstract

Choline is an essential nutrient in ruminant diets, which contributes to the fundamental biological functions of the animal. However, choline is easily degraded in the rumen before it can be absorbed. Rumen-protected choline (RPC) supplementation might support the fast growth of ruminants. This study aimed to investigate the effects of supplementing graded levels of RPC in a pelleted total mixed ration for fattening lambs. Sixty three-month-old male Small Tail Han and northeast fine wool sheep hybrid lambs with a liveweight of 15.3 ± 1.8 kg (mean ± SD) were fed designated diets and randomly assigned into five treatment groups (*n* = 12 per group). The five treatments were the rate of RPC supplementation at 0, 1.25, 2.50, 3.75, and 5.00 g (equivalent to 0, 0.31, 0.63, 0.94, and 1.25 g of choline chloride, respectively)/kg basal diet and the RPC-supplemented feed was offered for 112 days after 12 days of adaptation. Average daily gain, dry matter intake, and nutrient digestibility were similar across treatments. The rumen pH was quadratically significant among treatments, with the lowest and highest pH observed from the 2.5 and 5 g/kg RPC supplement groups, respectively (*P* = 0.02). After feeding, the ruminal ammonia concentrations among treatments were different (*P* < 0.05), with the highest value observed from the 5 g/kg RPC supplement group. Microbial crude protein level was different, with the highest value recorded from the 0 g/kg RPC supplement group (*P* = 0.028). A linear effect (*P* < 0.05) was observed from short-chain fatty acid values among treatments before and after feeding. Serum albumin (*P* = 0.003) and albumin/globulin ratio (*P* = 0.002) had a quadratic effect, with the highest value found in the 0 g/kg RPC supplement group. Abdominal fat was higher in RPC-supplemented groups (*P* < 0.05) compared to the control group. Drip loss was 65% higher in RPC-supplemented groups compared to the control group (*P* = 0.012). Overall, the study results showed an effect of RPC on ruminal parameters, but the supplementation of low-level RPC did not improve the growth and slaughter performance of fattening lambs.

## Introduction

Lamb fattening in some countries is shifting to adopting pelleted total mixed rations (PTMR) with a high proportion of cereals, which promotes a great growth rate and high economic return (1–4). One of the issues with this fattening practice is the excessive deposition of fat in the body, and consequently, the carcasses from these lambs have unfavorable sensory attributes for consumers. It is reported that choline can modulate lipid metabolism in the animal body (5), mainly because of the involvement of choline in lipid absorption and transportation (6). Particularly, choline can potentially promote the concentration of plasma low-density lipoprotein (LDL) cholesterol and the ratio of total cholesterol to high-density lipoprotein (HDL) cholesterol. Therefore, choline could reduce fat content in tissues and improve lamb meat quality (7, 8). In addition, choline was found to improve growth performance and carcass characteristics in beef cattle and lambs (7–10).

Choline is a vitamin-like essential nutrient, and the amount required by animals is as high as several orders of magnitude of other vitamins (11, 12). Choline is naturally present in feed ingredients at different concentrations (13). However, dietary choline is susceptible to microbial degradation in the rumen and limited amounts escape the rumen intact. Therefore, the supply of choline from feed to the small intestine is marginal and supplementation of choline provided in the rumen-protected form is recommended for ruminants (14).

In the current study it was hypothesized that the supplementation of rumen-protected choline (RPC) to fattening lambs fed PTMR would improve animal growth and reduce fat deposition in the body and the requirements for choline for lambs fed with PTMR would be different from those fed with other feeds. The objectives of this study were to determine growth rate, feed digestion, rumen fermentation characteristics, slaughter performance, meat quality, and serum metabolites when fattening lambs were fed PTMR supplemented with different amounts of RPC.

## Materials and methods

The research trial on animals was approved in advance by the Animal Ethics and Welfare Committee of Jilin Agricultural Science and Technology University, Jilin city, Jilin province, China (Approval Number 2019001) and conducted at the Animal Experimental Station of Jilin Agricultural Science and Technology University, Jilin City, Jilin Province, China.

### Experimental design and animals

The experiment included five dietary treatments, *i.e*., RPC supplementation at 0, 1.25, 2.5, 3.75, and 5 g/kg of the basal diet DM. The RPC supplement contained 25% choline chloride (Shandong Fulikang Animal Nutrition Co., Ltd, Binzhou, Shandong, China).

Eighty, brucellosis test negative (15), 3-month-old hybrid Small Tail Han and northeast fine wool rams were purchased for the experiment. After a period of 7-day dietary transition from hay to the pellet feed, 60 healthy lambs with no behavioral abnormalities of similar liveweights (averaging 15.3 ± 1.8 kg) were chosen and randomly allocated to one of five treatment groups, with 12 animals for each group. These lambs were further adapted to pellet feed for five more days and then fed the designated treatment diets with different amounts of RPC supplementation, starting on day 13 which was the first day of the measurement period. The formal experiment had three experimental periods: fattening period 1 for 56 days, fattening period 2 for another 56 days, and a digestibility measurement period for 10 days. At the end of the experiment, lambs from groups of RPC supplemented at 0 and 5 g/kg were slaughtered. From the start of the formal experiment, lambs were weighed before morning feeding every 4 weeks with an accuracy of 0.05 kg. Average daily gain (ADG) was estimated as the slope of liveweight against time.

### Feed and feeding

Lambs were fed with PTMR formulated according to the Chinese Feeding Standard for Lamb Finishing (16). The diets (Table 1) were pelleted at the Tongliao Subsidiary Company of Jiangsu Portal Agri-Industries Co., Ltd. Before pelleting, corn, cottonseed meal, sunflower meal, and soybean meal were ground and passed through a 2.0 mm mesh, and RPC was premixed with corn germ meal. After the ingredients were mixed, pelleting was performed using a pelleting machine (Model 400, Jiangsu Zhengchang Grain Machinery Co., Ltd., Liyang, Jiangsu, China). The pelleting conditions were: steam conditioning for 28 sec, pelleting at 82°C, counterflow cooling, ring die compression ratio 1:8, and ring die aperture 3.5 mm. The room temperature was 21.6°C. The pellets were 3.5 mm in diameter and 0.8–1.5 mm in length. Feed samples were collected during the two fattening periods and analyzed for dry matter (DM), crude protein (CP), neutral detergent fiber (NDF), acid detergent fiber (ADF), Ca, and P contents as described by Huo et al. (17).

**Table 1:** Ingredients and nutrient contents of experimental diets.

**Period**		**RPC suppement (g/kg)**				
		**0**	**1.25**	**2.5**	**3.75**	**5**
**1**	**Ingredients (g/kg as fed)**					
	Corn	500	500	500	500	500
	Premix^†^ (trace mineral salt and vitamins)	20	20	20	20	20
	Corn germ meal	100	99	98	96	95
	Sunflower seed meal	80	80	80	80	80
	Sunflower seed shell	44	44	44	44	44
	Corn stover	60	60	60	60	60
	Soybean meal	40	40	40	40	40
	Cottonseed meal	50	50	50	50	50
	Barley malt rootlets	80	80	80	80	80
	Limestone	16	16	16	16	16
	Calcium hydrogen phosphate	5	5	5	5	5
	Sodium chloride	5	5	5	5	5
	Rumen-protected choline (RPC)	0	1.25	2.5	3.75	5
	**Nutrient contents**^‡^ **(g/kg of DM**^**§**^**)**					
	DM (g/kg as fed)	945	923	924	944	925
	Crude protein (CP)	193	182	185	187	178
	Neutral detergent fiber (NDF)	242	260	252	265	247
	Acid detergent fiber (ADF)	127	123	125	132	122
	Ca	10.8	11.8	11.5	11.7	11.5
	P	5.6	5.2	5.1	5.2	5.1
**2**	**Ingredients**					
	Corn	600	600	600	600	600
	Premix	20	20	20	20	20
	Corn germ meal	142	141	140	138	137
	Sunflower seed meal	150	150	150	150	150
	Cottonseed meal	30	30	30	30	30
	Barley malt rootlets	30	30	30	30	30
	Limestone	15	15	15	15	15
	Calcium hydrogen phosphate	8	8	8	8	8
	Sodium chloride	5	5	5	5	5
	RPC	0	1.25	2.5	3.75	5
	**Nutrient contents**					
	DM (g/kg as fed)	944	946	944	933	943
	CP	160	164	161	162	161
	NDF	225	229	219	226	224
	ADF	100	92	92	91	87
	Ca	11.5	10.7	11.3	10.8	11.4
	P	5.9	5.8	5.8	5.9	5.7

† Premix per kg contained 200,000 IU vitamin A, 60,000 IU vitamin D_3_, 550 mg vitamin E, 800 mg nicotinamide, 650 mg Cu (as CuSO_4_), 2,800 mg Fe (as FeSO_4_), 900 mg Mn (as MnSO_4_), 16 mg Se (as Na_2_SeO_3_), 3,600 mg Zn (as ZnSO_4_), 20 mg Co (as CoCl_2_), 15 mg [as Ca(IO_3_)_2_], and 15 g lysine. The carrier was composed of glucose, rice bran, zeolite powder, and limestone powder.

‡ The nutrient contents were measured values.

§ DM = dry matter.

Lambs were kept in a semi-open shed covered with a plastic membrane and a sunshade net during the whole experimental period. During the 7-day dietary transition period, pellet feed was increased from 250 g/d per lamb with 50 g/d steps until only pellets were fed. Corn stover was offered *ad libitum* during this transition period. Pelleted feed was the sole feed for the rest of the experimental periods. The feed was provided twice daily with equal portions at 08:00 and 15:00 h, and water was given *ad libitum*. Feed allowance was adjusted based on the previous intake to allow refusal of around 10% of feed offered. Lambs were orally dosed with albendazole at 15 mg per kg of live weight for deworming prior to each of the two fattening periods. Weather, temperature, and humidity were recorded daily, and animal behavior was observed for animal welfare concerns.

### Digestibility measurements

The measurements of apparent total tract digestibility were conducted in metabolic cages using the total fecal collection method (18). The digestibility period lasted for 10 days, including 4 days for lambs to adapt to the cage housing conditions and wearing the fecal collection harness and 6 days for total fecal matter collection. Lambs were fed individually, and feed allowance was adjusted to allow 5–10% of the feed to be left. Feed refusals and feces were collected and quantified daily. These samples were processed and stored as described by Huo et al. (17). Feed, refusals and feces samples were dried at 65°C for 48 h, ground, and analyzed for DM, NDF, and ADF as described above.

### Analyses of blood and rumen samples

On day 12 of fattening period 2, blood (5 ml) was collected from the jugular vein of each lamb before morning feeding. The blood was collected into a coagulation promoting tube with separating gel (Sanli Industrial Co., Ltd., Huizhou, China). The collected blood samples were centrifuged at 1,000 × *g* for 10 min (Model TDL-80-2B; Anting Scientific Instrument Factory, Shanghai, China) and the serum analyzed for blood biochemical parameters using an automatic biochemical analyser (Model 7160; Hitachi Ltd., Tokyo, Japan). Blood biochemical parameters assayed included total protein, albumin, globulin, blood urea nitrogen (BUN), creatinine, glucose, triglyceride, cholesterol, high-density lipoprotein cholesterol (HDL), low-density lipoprotein cholesterol (LDL), alanine transaminase (ALT), aspartate transaminase (AST), alkaline phosphatase (ALP), direct bilirubin (DBIL), γ-glutamyl transpeptidase (GGT), and total bilirubin (TBIL).

Rumen contents were taken using an esophageal tube before morning feeding on day 37 of fattening period 1 and 2.5 h after morning feeding the next day. The first 50 ml sample was discarded to minimize saliva contamination and the second 50 ml sample was kept for analysis. The pH value was immediately measured using a pH meter with an accuracy of 0.01 pH units (LICHEN pH-100A, Shanghai Lichen Scientific Laboratory Instrument Ltd., Shanghai, China). Then the sample was transferred into 2-ml cryogenic vials (Corning Inc., New York, USA) and stored at −20°C. After thawing in a stream of running tap water, the sample was centrifuged at 10,000 × *g* for 10 min at 4°C. Then the supernatant (1 mL) was harvested and transferred to a tube containing 0.2 mL 25% (w/w) metaphosphoric acid and 60 mM crotonic acid as an internal standard and centrifuged at 10,000 × *g* at 4°C for 10 min. The supernatant was used to determine ammonia concentration using a modified Indigo phenol blue-spectrophotometry method (19), and short-chain fatty acids (SCFA) using gas chromatography (Model GC-9A; Shimadzu Co., Japan) coupled with a flame ionization detector (FID) and a polar capillary column (CP-WAX, 30 m × 0.53 mm × 1 μm). The settings of the gas chromatography conditions were a starting column temperature of 100°C increasing at 3°C per min and ending at 150°C. The FID was set at 200°C, the vaporization chamber was set at 200°C, N_2_ was used as the carrier gas and the injection volume was 0.4 μL. Microbial crude protein (MCP) was quantified using spectrophotometric and HPLC methods described by Zinn and Owens (20) and modified by Makkar and Becker (21) with yeast RNA as a standard.

### Slaughter and meat quality measurements

Seven lambs from the control group and seven from the 5 g/kg RPC group were randomly chosen for slaughter. After 24 h fasting, slaughtering was conducted by cutting blood vessels, trachea, and esophagus. This was carried out by a single person to minimize experimental errors. After slaughter, fat in the abdomen, kidney, and pelvic cavity were collected and the weights were recorded. The carcass was weighed within 0.5 h and recorded as hot carcass weight (HCW). Then the carcass was halved, with the left half for the determination of meat color, marbling, and pH. Within 1 h after slaughter, fresh meat samples were taken from the cross-section of the upper eye muscle between the penultimate rib and the second rib.

Inside a room under natural light, meat color and marbling were scored by visual comparison with the American standard color and marbling scoring cards for pork. The pH values in the shoulder, *longissimus dorsi*, and gluteus muscles were determined at room temperature using a pH probe (Model pH-STAR; Matthäus GmbH, Poettmes, Germany) within 45 min after slaughter with the average of triplicate readings recorded. The measurement was conducted by inserting the pH probe into the meat at a depth of 15 mm. Then, about 200 g of the shoulder, back, and buttock muscles were collected for the determination of cooking loss, water loss, drip loss, tenderness, and fat content as described below.

Fascia and attached fat were removed from the middle section of the *longissimus* muscle. Within 12 h post-mortem, the meat sample was steamed for 30 min. After cooling, the meat sample was weighed. The cooked meat rate was calculated as the percentage of the meat weight after cooking relative to the meat weight before cooking. The water loss rate of the *longissimus* muscle was measured using the filter paper press method of Grau and Hamm (22). The *longissimus dorsi* muscle between the 3^rd^ and 4^th^ rib was cut into pieces that were 5 cm in length, 3 cm in width, and 2.5 cm in height. The meat piece was weighed, placed in an aerated Polybrene bag and suspended in a freezer at 4°C. After 24 h, the meat piece was weighed again after water from the surface was swabbed. Drip loss was calculated as the percentage of weight loss. The meat sample with fat removed from the surface was put in a self-sealed bag and kept at 4°C for 24 h. Then the sample was left at room temperature for 1 h and cooked at 80°C in a thermostatic water bath until the center of the meat reached 70°C. After cooling down to room temperature, the meat sample was sliced to 1.5 cm thickness. The tenderness [expressed as Newton (N)] was determined using a circular sampler with a diameter of 1.27 cm. The average was obtained from three readings for each meat sample. Fat content was measured using the Soxhlet extraction method (AOAC 991.36) and expressed as the weight percentage of wet muscle.

### Statistical analysis

Data of animal performance, digestibility, rumen fermentation characteristics and blood biochemical parameters were checked for normal distribution and analyzed with the GLM procedure for linear and quadratic responses to RPC supplementation level using GenStat 21st edition (VSN International, Hemel Hempstead, UK, 2021) (23). Animal performance was separately analyzed for the two fattening periods. Slaughter performance and meat quality of the 0 and 5 g/d RPC were analyzed using one-way ANOVA. Duncan's test was conducted for multiple treatment comparisons. The significance of difference was declared at *P* < 0.05 and tendency at 0.05 < *P* < 0.10.

## Results

### Growth performance and digestibility

The initial, middle and final body weights of lambs among the treatments were similar. The ADG did not show a significant difference among the treatments at any time (Table 2).

**Table 2:** Growth performance of fattening lambs fed experimental diets supplemented with nil (CON) or rumen-protected choline (RPC) (*n* = 12 per treatment).

**Item**	**RPC supplement (g/kg)**					**SEM^†^**	***P*** **-value**	
	**0**	**1.25**	**2.5**	**3.75**	**5**		**Linear**	**Quadratic**
Initial BW^‡^ (d 0, kg)	15.5	15.3	15.2	15.3	15.5	0.55	0.994	0.683
Middle BW (d 56, kg)	29.5	30.5	30.9	27.1	30.5	0.95	0.662	0.878
Final BW (d 112, kg)	43.2	45.0	43.0	41.5	43.4	1.49	0.518	0.900
ADG^§^ (period 1, d 0–56, g/d)	252	274	279	211	269	15.7	0.565	0.979
ADG (period 2, d 56–112, g/d)	270	249	216	257	241	23.3	0.501	0.335
Overall ADG (d 0–112, g/d)	246	259	242	234	249	11.4	0.606	0.765

† SEM, standard error of the mean.

‡ BW, body weight.

§ ADG, average daily gain.

There was no significant difference in dry matter intake (DMI) and digestibility of DM and other nutrients among treatments (Table 3). However, the NDF digestibility tended (*P* = 0.08) to be significant increased when the RPC supplement was increased.

**Table 3:** Intake and total tract apparent nutrient digestibility of fattening lambs fed experimental diets supplemented with nil (CON) or rumen-protected choline (RPC) (*n* = 6 per treatment).

**Item**	**RPC supplement (g/kg)**					**SEM^†^**	***P*** **-value**	
	**0**	**1.25**	**2.5**	**3.75**	**5**		**Linear**	**Quadratic**
DM intake (g/d)	986	847	904	893	929	50.2	0.668	0.146
**Digestibility (g/g of DM)**								
DM^‡^	0.719	0.727	0.717	0.725	0.722	0.0146	0.924	0.959
CP^‡^	0.734	0.717	0.716	0.743	0.714	0.0152	0.758	0.934
NDF^‡^	0.320	0.268	0.315	0.389	0.370	0.0384	0.080	0.518
ADF^‡^	0.245	0.222	0.256	0.338	0.281	0.0448	0.195	0.905

† SEM, standard error of the mean.

‡ DM, dry matter; OM, organic matter; CP, crude protein; NDF, neutral detergent fiber; ADF, acid detergent fiber.

### Rumen fermentation and blood biochemical parameters

The rumen pH measured 2.5 h after the morning feeding was quadratically significant (*P* = 0.02) among treatments, with the lowest and highest pH observed for the 2.5 and 5 g/kg RPC supplement groups, respectively. Before the morning feeding, rumen ammonia (NH_3_) concentration was not significantly different among the treatments. However, after feeding, the NH_3_ concentrations among groups were significantly different (*P* < 0.05), with the highest value observed in the 5 g/kg RPC supplement group (Table 4).

**Table 4:** Rumen fermentation parameters of fattening lambs fed experimental diets supplemented with nil (CON) or rumen-protected choline (RPC) (*n* = 6 per treatment).

**Sampling time**	**Item**	**RPC supplement (g/kg)**					**SEM^†^**	***P-v*** **alue**	
		**0**	**1.25**	**2.5**	**3.75**	**5**		**Linear**	**Quadratic**
**Before** ^‡^	pH	7.04	6.43	6.82	6.94	6.52	0.159	0.081	0.472
	NH_3_ (mM)	9.8	9.1	11.6	10.7	11.0	1.06	0.250	0.745
	MCP^§^ (g/L)	4.37	8.13	5.50	6.27	5.84	0.876	0.721	0.140
	Total SCFA^§^ (mM)	27.7^a^	71.0^c^	44.3^abc^	40.1^ab^	63.5^bc^	6.65	0.047	0.549
	**Molar proportion in the total SCFA (mol/100 mol)**								
	Acetate	57.7^b^	51.2^a^	48.6^a^	50.7^a^	51.2^a^	1.26	0.003	<0.001
	Propionate	28.7^a^	38.0^b^	41.6^b^	38.5^b^	39.6^b^	1.54	<0.001	<0.001
	Butyrate	5.5	6.7	4.6	4.8	5.0	0.89	0.285	0.892
	*iso*-Butyrate	2.5^b^	0.8^ab^	1.1^ab^	1.7^ab^	0.5^a^	0.47	0.034	0.507
	Valerate	2.8	2.5	2.2	2.4	2.5	0.28	0.486	0.240
	*iso*-Valerate	2.8^b^	0.8^a^	1.9^ab^	1.9^ab^	1.3^a^	0.33	0.047	0.206
	Acetate: propionate	2.02^b^	1.37^a^	1.17^a^	1.33^a^	1.33^a^	0.084	<0.001	<0.001
**After** ^‡^	pH	5.67^ab^	5.74^ab^	5.44^a^	5.54^ab^	6.03^b^	0.136	0.166	0.020
	NH_3_ (mM)	5.94^a^	6.51^a^	6.29^a^	5.48^a^	9.32^b^	0.477	<0.001	0.002
	MCP (g/L)	5.18^b^	3.79^ab^	3.41^ab^	4.36^ab^	2.86^a^	0.580	0.028	0.642
	Total SCFA (mM)	104.9^b^	102.5^ab^	96.0^ab^	103.0^ab^	83.5^a^	5.50	0.015	0.294
	**Molar proportion in the total SCFA (mol/100 mol)**								
	Acetate	43.4	46.7	48.7	45.8	47.9	1.94	0.197	0.324
	Propionate	43.3	42.9	41.7	44.1	42.0	3.22	0.879	0.996
	Butyrate	9.7	7.2	6.1	7.1	6.3	1.48	0.149	0.332
	*iso*-butyrate	0.2	0.2	0.3	0.0	0.2	0.12	0.931	0.985
	Valerate	3.2	2.5	2.5	2.5	2.9	0.51	0.760	0.283
	*iso*-valerate	0.3	0.5	0.7	0.4	0.8	0.17	0.105	0.783
	Acetate: propionate	1.06	1.17	1.19	1.07	1.21	0.144	0.623	0.883

† SEM, standard error of the mean.

‡ Before, before the morning feeding; After, 2.5 h after the morning feeding;

§ MCP, microbial crude protein; SCFA, short chain fatty acids. The letters with different superscripts differ significantly with values of *P* < 0.05.

Microbial crude protein level was similar among treatments before the morning feeding but was significantly different at 2.5 h after the morning feeding, with the highest value recorded for the 0 g/kg RPC supplement group (*P* = 0.028). A linear effect (*P* < 0.05) was observed for SCFA values among treatments before and after feeding, however, SCFA increased linearly pre-feeding and decreased linearly post-feed with increasing RPC level. A significant linear effect (*P* < 0.05) was observed for acetate, propionate, *iso*-butyrate, *iso*-valerate, and acetate: propionate ratio before feeding, while there were no significant effects detected for these parameters after feeding (Table 4).

Serum albumin (*P* = 0.003) and A/G (*P* = 0.002) had a quadratic effect, with the highest value found in the 0 g/kg RPC supplement group. Globulin and LDL had a linear effect, with the lowest value found in the 0 g/kg RPC supplement group (*P* < 0.05; Table 5). The ALT and ALP from the liver function panel had a quadratic effect, with the highest value found in 1.25 and 0 g/kg RPC supplement groups, respectively. The values of uric acid had shown a linear effect (*P* < 0.05), with the highest value found in the 5 g/kg RPC supplemented group. RPC level had no significant effect on total protein, BUN, creatinine, triglyceride, cholesterol, HDL, AST, TBIL and GGT.

**Table 5:** Serum biochemical parameters of fattening lambs fed experimental diets supplemented with nil (CON) or rumen-protected choline (RPC) (*n* = 6 per treatment).

**Item**	**RPC supplement (g/kg)**					**SEM^†^**	***P-*** **value**	
	**0**	**1.25**	**2.5**	**3.75**	**5**		**Linear**	**Quadratic**
**Protein metabolism**								
Total protein (g/L)	66.6	62.8	65.7	66.0	65.7	1.65	0.781	0.496
Albumin (A) (g/L)	40.0^b^	31.9^a^	33.7^a^	36.6^ab^	35.5^ab^	1.22	0.271	0.003
Globulin (G) (g/L)	29.4^a^	33.2^b^	32.7^ab^	32.5^ab^	33.2^b^	1.00	0.040	0.120
A/G^‡^	1.37^b^	0.97^a^	1.03^a^	1.13^a^	1.08^a^	0.056	0.024	0.002
BUN^‡^ (mmol/L)	10.6	9.3	10.8	10.5	10.0	0.19	0.833	0.913
Creatinine (μmol/L)	42.1	44.8	46.9	41.5	47.6	1.70	0.154	0.908
**Energy substrates and enzymes**								
Glucose (mmol/L)	4.40^a^	5.20^b^	4.91^ab^	4.70^ab^	4.66^ab^	0.137	0.961	0.004
Triglyceride (μmol/L)	0.24	0.40	0.27	0.28	0.30	0.040	0.995	0.362
Cholesterol (mmol/L)	1.26	1.56	1.49	1.80	1.45	0.150	0.202	0.116
HDL^‡^ (mmol/L)	0.78	0.74	0.69	0.85	0.68	0.057	0.568	0.800
LDL^‡^ (mmol/L)	0.42^a^	0.74^ab^	0.74^ab^	0.88^b^	0.71^ab^	0.109	0.045	0.051
**Liver function**								
ALT^‡^ (U/L)	12.9^a^	18.3^c^	14.4^abc^	17.2^bc^	13.5^ab^	0.99	0.987	0.006
AST^‡^ (U/L)	7.58	7.08	8.17	6.82	8.13	0.725	0.718	0.663
ALP^‡^ (U/L)	593	425	426	336	488	65.2	0161	0.034
AST/ALT^‡^	0.515	0.390	0.537	0.478	0.568	0.0831	0.466	0.475
TBIL^‡^ (μmol/L)	0.75	0.40	0.35	0.53	0.65	0.164	0.885	0.068
GGT^‡^ (U/L)	6.73	3.38	4.38	3.12	4.35	1.203	0.199	0.138
DBIL^‡^ (μmol/L)	0.33	1.43	0.40	0.64	0.47	0.255	0.519	0.194
Uric acid (μmol/L)	2.4^ab^	2.0^a^	2.6^ab^	2.4^ab^	3.5^b^	0.53	0.035	0.117

† SEM, standard error of the mean.

‡ A/G, albumin/globulin ratio; ALP, alkaline phosphatase; ALT, alanine transaminase; AST, aspartate transaminase; AST/ALT, aspartate transaminase/alanine transaminase ratio; BUN, blood urea nitrogen; DBIL, direct bilirubin; GGT, γ-glutamyl transpeptidase; HDL, high density lipoprotein cholesterol; LDL, low density lipoprotein cholesterol; TBIL, total bilirubin. The letters with different superscripts differ significantly with values of *P* < 0.05.

### Slaughter performance and meat quality

The supplementation of RPC had no significant effect on most parameters examined in terms of slaughter performance and meat quality of fattening lambs (Table 6). However, the supplementation of RPC tended to increase HCW (*P* = 0.097) and dressing percentage (*P* = 0.066). Furthermore, abdominal fat was significantly (*P* < 0.05) higher in RPC-supplemented group compared to the control group and drip loss was 65% higher in RPC-supplemented group compared to the control group (*P* = 0.012). The fat content in the hip muscle was lower (*P* < 0.05) in the treatment group compared to the control group.

**Table 6:** Slaughter performance and meat quality of fattening lambs fed experimental diets supplemented with nil or rumen-protected choline (RPC) (*n* = 7 per treatment).

**Item**	**RPC supplement (g/kg)**		**SEM^†^**	***P*-value**
	**0**	**5**		
Live weight (kg)	46.7	50.8	1.85	0.168
Hot carcass weight (HCW, kg)	20.4	23.0	0.99	0.097
Dressing percentage (%)	43.6	45.3	0.57	0.066
**Fat**				
Kidney fat (g)	215	352	49.5	0.086
Pelvic fat (g)	55.2	39.7	12.7	0.427
Abdominal fat (g)	268^a^	455^b^	57.0	0.048
Kidney fat/HCW (%)	0.46	0.67	0.080	0.101
Pelvic fat/HCW (%)	0.12	0.08	0.025	0.297
Abdominal fat/HCW (%)	0.57	0.89	0.107	0.066
**Meat quality**				
Marbling score	1.00	1.14	0.101	0.337
Meat color	5.86	5.86	0.143	1.000
Cooked meat percentage (%)	54.3	53.6	0.49	0.315
Water loss rate (%)	39.7	40.6	1.55	0.703
Drip loss (%)	2.81^a^	4.65^b^	0.414	0.012
Tenderness (N)	41.4	42.3	3.97	0.873
Shoulder muscle pH	6.92	6.60	0.154	0.162
Back muscle pH	6.33	6.43	0.153	0.654
Hip muscle pH	6.38	6.39	0.121	0.944
Fat content in shoulder muscle	5.25	7.09	0.81	0.151
Fat content in back muscle	6.39	6.42	0.84	0.980
Fat content in hip muscle	5.97^b^	4.09^a^	0.53	0.026

† SEM, standard error of the mean.

## Discussion

The DMI was similar among treatments, and in the literature the effect of supplementing RPC on DMI has been inconsistent. For example, studies on goats (24, 25) noted an increase in DMI when supplemented with RPC, whereas studies with sheep (7, 8, 26) reported no effect of RPC on DMI. The effect of RPC supplementation on DMI could be influenced by many factors, such as choline purity, amount of RPC supplement offered, animal state and rumen protection rate of choline. It was found that RPC supplemented to the diet of dairy cows can be derived to trimethylamine *via* rumen microbial metabolism and further converted to trimethylamine N-oxide (27), while trimethylamine-N-oxide was associated with depressed DMI in some studies (28), but not in other studies (29). The use of RPC in the current study may have ameliorated the negative effects of free choline on DMI resulting in similar DMI among treatments. However, some previous studies have shown that RPC has the potential to enhance appetite in ruminants (30, 31), which was not observed in the current study.

Previous studies have shown that the supply of RPC promotes feed digestion and nutrient absorption, and increases the overall digestibility of feed in lambs (31). However, increasing the level of RPC from 0 to 5 g/kg in the basal diet of lambs in the present study did not have a significant effect on apparent total tract digestibility, which also was the case in a previous study with lambs (26) and dairy cows (32). The possible reasons for the lack of a response in digestibility may be that the highest level of RPC supplementation in our study of 5 g/kg is 20% lower than in the study of Li et al. (31) who observed an increase in apparent digestibility when lambs were supplemented with RPC at a level of 7.5 g/kg. Interestingly, the supplementation of RPC tended to increase NDF digestibility in the present study. Arce-Cordero et al. (33) found that adding unprotected choline chloride to ruminal dual-flow continuous-culture fermenters decreased the abundance of fiber-degrading bacteria, from which a decrease in NDF digestibility would be expected. The choline we used in the present study is rumen-protected. This choline can be partially released in the rumen. The amount of released choline was obviously not enough to have such an effect to decrease fiber degradation and in contrast, NDF digestibility tended to increase. We do not have an explanation for this increase, which warrants further studies.

Supplementation of RPC did not affect ADG in the present study. Similarly, a recent study by Kawas et al. (26) also noted no effect of RPC supplementation on the growth performance of Saint Croix lambs. In contrast, a study by Li et al. (7) reported statistically higher ADG of 211 g/d of lambs supplemented with 0.25% RPC compared to lambs supplemented with 0% (186 g/d), 0.5% (178 g/d) and 0.75% (170 g/d) RPC. In another study on lambs, ADG was 167, 191, and 188 g/d when supplemented with 0, 0.25, and 0.75% RPC, respectively (34). Similarly, Pinotti et al. (10) found that supplementing beef cattle with RPC increased ADG. Bryant et al. (8) found an improved growth performance of RPC-supplemented steers and lambs. Kawas et al. (26) also noted the inconsistency in animal growth performance in response to RPC supplementation among studies and they suggested that this inconsistency might be attributed to factors, such as dietary protein level, the proportion of grain in the diet and animal breed. These factors may also explain some of the differences between our study and the study by Li et al. (7), for example, the different levels of RPC supplementation, a higher concentration of CP (over 16%) in our study compared to 12% in the study by Li et al. (7) and different breeds used in the two studies. In the first period of the experiment, CP contents ranged from 178 to 193 g/kg of DM among treatments. These contents were higher than animal requirements for CP recommended by NRC (11). Thus CP contents in the diet were not a limiting factor for growth and consequently lamb growth was not affected by RPC supplementation. In addition, our study used high energy PTMR, while the effect of RPC was previously found to be greater when supplemented into low-energy diets (7, 25).

In the current study, the 2.5 g RPC/kg supplement group was the only group with a pH value below 5.5 at 2.5 h after the morning feeding. The reason for this low pH in the 2.5 g/kg supplement group is not clear. In a recent study by Leal et al. (35) supplementing lambs with biocholine powder had no effects on rumen pH either. However, we used RPC as a source of choline, whereas biocholine was used in the study of Leal et al. (35).

In our study, MCP level was similar among treatments before feeding, and they became significantly different 2.5 h after the morning feeding, with the highest value (5.2 g/L) recorded for the 0 g/kg RPC supplement group and the lowest value (2.9 g/L) recorded for the 5 g/kg supplement group. Possible reasons for the low concentration of MCP in the RPC-supplemented group in the current study are unclear but might be due to partial degradation of RPC by rumen microbes in the rumen. To our knowledge, no other study has determined the effect of RPC on MCP. Therefore, this effect needs further investigation.

As observed in the study by Li et al. (7), the supplementation of RPC had little effect on blood lipids, except the LDL concentration increased with higher RPC levels. The increase in LDL concentration with high levels of RPC may be due to the synthesis of phosphatidylcholine, which is enhanced by the supply of choline (36, 38). The effects of adding choline to the diet on serum triglycerides have been inconsistent (7, 26, 37). For example, Mohsen et al. (37) found that RPC supplementation led to a significant decrease in the concentrations of plasma cholesterol and triglycerides. However, similar to the study of Li et al. (7), we did not find an association between RPC supplementation and the concentrations of serum total triglycerides and cholesterol. The variation in response may be due to the different animal breeds, diets, and physiological stages (7).

Most slaughter performance and meat quality parameters of fattening lambs fed 0 or 5 g/kg were similar in this study but HCW and dressing percentage tended to increase. This is consistent with findings of studies by Li et al. (7) and Kawas et al. (26) who also reported no effects of RPC supplementation on slaughter performance and carcass characteristics of lambs. In contrast, Dong et al. (39) recorded less abdominal fat with 2.2 g/d rumen-protected betaine (RPB) and abdominal fat was higher in the 5 g/kg supplemented group compared to the un-supplemented group. The difference between this and our study might be because our study used RPC whereas the study of Dong et al. (39) used RPB, and choline needs to be further oxidated into betaine in the mitochondrion before metabolism (40). Drip loss was 65% higher in the 5 g/kg supplemented group than in the control group in the current study. This is not a desirable result because excessive drip losses not only cause financial losses but also result in losses in valuable vitamins, minerals, flavor compounds and water which can affect overall eating quality, producing meat that can be described as tough, and having poor mouthfeel characteristics (41). In contrast, Li et al. (7) found drip loss was smaller in the RPC supplemented lambs than in the un-supplemented lambs. More studies are needed to clarify this discrepancy.

As discussed above, there were some biological effects of RPC when it was supplemented to PTMR for fattening lambs. However, the levels of RPC supplementation in the current study did not result in a significant improvement in animal growth and slaughter performance. Therefore, supplementation of RPC at levels used in the current study cannot be recommended for fattening lambs fed PTMR.

## Conclusion

In conclusion, supplementation of RPC at 0, 1.25, 2.5, 3.75, and 5 g/kg did not affect lamb growth performance but tended to increase HCW and dressing percentage. Rumen MCP concentrations were similar among treatments before feeding, and they became significantly different 2.5 h after the morning feeding, with the highest value recorded for the control without RPC supplementation. The results of the current study suggest that there is little benefit when supplementing 5 g/kg or less RPC in the PTMR diet of fattening lambs.

## Data availability statement

The data that support the findings of this study are available on request from the corresponding author.

## Ethics statement

The animal study was reviewed and approved by Animal Ethics and Welfare Committee, Jilin Agricultural Science and Technology University (Approval number 2019001).

## Author contributions

XS and PY conceived and planned the study. XS acquired funding, supervised all research, analyzed and interpreted data, prepared the tables, and wrote the early version of the manuscript. XS, QH, PY, and MY organized resources. QH and TW conducted the animal experiment and analyzed the samples. QH, TW, RL, JL, WT, CL, CW, YH, and XS collected samples. XS, ZL, AJ, IR, and LC reviewed and edited the manuscript. All authors approved the final version of the manuscript.

## Funding

This study was funded by the Department of Science and Technology of Jilin Province, China (Grant Number 20220202052NC).

## Conflict of interest

Authors PY and MY were employed by Portal Agri-Industries Co., Ltd. Author IR was employed by Lely Australia Pty Ltd. The remaining authors declare that the research was conducted in the absence of any commercial or financial relationships that could be construed as a potential conflict of interest.

## Publisher's note

All claims expressed in this article are solely those of the authors and do not necessarily represent those of their affiliated organizations, or those of the publisher, the editors and the reviewers. Any product that may be evaluated in this article, or claim that may be made by its manufacturer, is not guaranteed or endorsed by the publisher.

## References

[1] Sun X Song B He Y You P. A review on pelleted complete feed for sheep and goats. Mod J Anim Husb Veterin Med. (2017) 46:162–5.

[2] Li B Sun XZ Huo Q Zhang GG Wu TT You PH. Pelleting of a total mixed ration affects growth performance of fattening lambs. Front Veterin Sci. (2021) 8:629016. 10.3389/fvets.2021.629016 33681330 PMC7928353

[3] Retnani Y Risyahadi ST Qomariyah N Barkah NN Taryati T Jayanegara A. Comparison between pelleted and unpelleted feed forms on the performance and digestion of small ruminants: a meta-analysis. J Anim Feed Sci. (2022) 31:97–108. 10.22358/jafs/149192/2022

[4] Sohail MA Rashid MA Habib HF Malik MI Yousaf MS Rehman H. Effects of physical form and wheat straw level in the diet on growth performance, nutrient digestibility, rumen papillae morphometry, and carcass characteristics in Lohi lambs. Anim Product Sci. (2022). 10.1071/AN21559

[5] Zenobi MG Scheffler TL Zuniga JE Poindexter MB Campagna SR Castro Gonzalez HF. Feeding increasing amounts of ruminally protected choline decreased fatty liver in nonlactating, pregnant Holstein cows in negative energy status. J Dairy Sci. (2018) 101:5902–23. 10.3168/jds.2017-13973 29680650

[6] Lagace TA. Phosphatidylcholine: greasing the cholesterol transport machinery. Lipid Insights. (2015) 8:65–73. 10.4137/lpi.S31746 27081313 PMC4821435

[7] Li H Wang H Yu L Wang M Liu S Sun L. Effects of supplementation of rumen-protected choline on growth performance, meat quality and gene expression in *longissimus dorsi* muscle of lambs. Arch Anim Nutr. (2015) 69:340–50. 10.1080/1745039X.2015.1073001 26305383

[8] Bryant TC Rivera JD Galyean ML Duff GC Hallford DM Montgomery TH. Effects of dietary level of ruminally protected choline on performance and carcass characteristics of finishing beef steers and on growth and serum metabolites in lambs. J Anim Sci. (1999) 77:2893–903. 10.2527/1999.77112893x 10568457

[9] Martínez-Aispuro JA Mendoza GD Cordero-Mora JL Ayala-Monter MA Sánchez-Torres MT Figueroa-Velasco JL. Evaluation of an herbal choline feed plant additive in lamb feedlot rations. Revista Brasileira de Zootecnia. (2019) 48:2. 10.1590/RBZ4820190020

[10] Pinotti L Paltanin C Campagnoli A Cavassini P Dell'Orto V. Rumen protected choline supplementation in beef cattle: effect on growth performance. Italian J Anim Sci. (2009) 8:322–4. 10.4081/ijas.2009.s2.322

[11] NRC. Nutrient Requirements of Small Ruminants: Sheep, Goats, Cervids and New World Camelids. 6th ed. Washington, DC: National Academy Press (2007). 384 p.

[12] Moretti A Paoletta M Liguori S Bertone M Toro G Iolascon G. Choline: an essential nutrient for skeletal muscle. Nutrients. (2020) 12:1–11. 10.3390/nu12072144 32708497 PMC7400816

[13] Rychen G Aquilina G Azimonti G Bampidis V Bastos ML Bories G. Safety and efficacy of betaine anhydrous for food-producing animal species based on a dossier submitted by AB Vista. EFSA J. (2018) 16:e05335. 10.2903/j.efsa.2018.5335 32625971 PMC7009651

[14] Jayaprakash G Sathiyabarathi M Robert MA Tamilmani T. Rumen-protected choline: a significance effect on dairy cattle nutrition. Veterin World. (2016) 9:837–41. 10.14202/vetworld.2016.837-841 27651671 PMC5021832

[15] Alton GG Jones LM Pietz DE. Laboratory Techniques in Brucellosis. (2nd ed.). Geneva: World Health Organization (1975). 1–163 p. 812265

[16] Ministry of Agriculture of China. Feeding Standard of Meat-Producing Sheep and Goats (Standard NY/T 816-2004). Beijing: Chinese Agricultural Press (2005).

[17] Huo Q Li B Cheng L Wu T You P Shen S. Dietary supplementation of lysophospholipids affects feed digestion in lambs. Animals. (2019) 9:805. 10.3390/ani9100805 31618894 PMC6826496

[18] Sun XZ Krijgsman L Waghorn GC Kjestrup H Koolaard J Pacheco D. Sheep numbers required for dry matter digestibility evaluations when fed fresh perennial ryegrass or forage rape. Anim Nutr. (2017) 3:61–6. 10.1016/j.aninu.2016.12.001 29767140 PMC5941065

[19] Feng Z Gao M A. Modified spectrophotometric method for the determination of ammonia concentration in ruminal liquor. Anim Husband Feed Sci. (2010) 31:37. 10.16003/j.cnki.issn1672-5190.2010.z1.027

[20] Zinn RA Owens FN A. Rapid procedure for purine measurement and its use for estimating net ruminal protein synthesis. Can J Anim Sci. (1986) 66:157–66. 10.4141/cjas86-017

[21] Makkar HPS Becker K. Purine quantification in digesta from ruminants by spectrophotometric and HPLC methods. Br J Nutr. (1999) 81:107–12. 10.1017/S0007114599000227 10450327

[22] Barge MT Destefanis G Toscano GP Brugiapaglia A. Two reading techniques of the filter paper press method for measuring meat water-holding capacity. Meat Sci. (1991) 29:183–9. 10.1016/0309-1740(91)90065-X 22061104

[23] VSN International. Genstat for Windows (21st Ed.) Web page: Genstat.co.uk ed. Hemel Hempstead: VSN International (2021).

[24] Tu YL Zhang K Bai YF Gao LP Hong W. Effects of rumen-protected choline supplied at different dietary energy levels on growth performance and meat quality of fattening goats. J Anim Feed Sci. (2020) 29:234–40. 10.22358/JAFS/127693/2020

[25] Habeeb AAM Gad AE Atta MAA Abdel-Hafez MAM. Evaluation of rumen-protected choline additive to diet on productive performance of male Zaraibi growing goats during hot summer season in Egypt. Trop Anim Health Prod. (2017) 49:1107–15. 10.1007/s11250-017-1292-x 28497207

[26] Kawas JR Garcia-Mazcorro JF Fimbres-Durazo H Ortega-Cerrilla ME. Effects of rumen-protected choline on growth performance, carcass characteristics and blood lipid metabolites of feedlot lambs. Animals. (2020) 10:1–9. 10.3390/ani10091580 32899809 PMC7552332

[27] Swartz TH Bradford BJ Malysheva O Caudill MA Mamedova LK Estes KA. Effects of dietary rumen-protected choline supplementation on colostrum yields, quality, and choline metabolites from dairy cattle. JDS Commun. (2022) 3:296–300. 10.3168/jdsc.2021-0192 36338018 PMC9623657

[28] Winther SA Rossing P. TMAO: Trimethylamine-N-oxide or time to minimize intake of animal products? J Clin Endocrinol Metabol. (2020) 105:111. 10.1210/clinem/dgaa428 32701146

[29] Myers WA Wang F Chang C Davis AN Rico JE Tate BN. Intravenous trimethylamine N-oxide infusion does not modify circulating markers of liver health, glucose tolerance, and milk production in early-lactation cows. J Dairy Sci. (2021) 104:9948–55. 10.3168/jds.2021-20169 34176629

[30] Zahra LC Duffield TF Leslie KE Overton TR Putnam D LeBlanc SJ. Effects of rumen-protected choline and monensin on milk production and metabolism of periparturient dairy cows. J Dairy Sci. (2006) 89:4808–18. 10.3168/jds.S0022-0302(06)72530-9 17106112

[31] Li H Wang H Wang M Yu L Sun L Chen Q. Effects of rumen protected choline on growth, digestion, serum indices and meat quality of lambs. Chin J Anim Nutr. (2015) 27:1117–23. 10.3969/j.issn.1006-267x.2015.04.015 26305383

[32] Nunes AT Takiya CS da Silva GG Ghizzi LG Grigoletto NTS Dias MSS. Increasing doses of biocholine on apparent digestibility, ruminal fermentation, and performance in dairy cows. Livestock Sci. (2022) 260:35. 10.1016/j.livsci.2022.104927

[33] Arce-Cordero JA Fan P Monteiro HF Dai X Jeong KC Faciola AP. Effects of choline chloride on the ruminal microbiome at 2 dietary neutral detergent fiber concentrations in continuous culture. J Dairy Sci. (2022) 105:4128–43. 10.3168/jds.2021-21591 35282921

[34] Jin Y Li H Wang H. Dietary rumen-protected choline supplementation regulates blood biochemical profiles and urinary metabolome and improves growth performance of growing lambs. Anim Biotechnol. (2021). 10.1080/10495398.2021.1984247 34658301

[35] Leal KW Alba DF Cunha MG Marcon H Oliveira FC Wagner R. Effects of biocholine powder supplementation in ewe lambs: growth, rumen fermentation, antioxidant status, and metabolism. Biotechnol Rep. (2021) 29:12. 10.1016/j.btre.2020.e00580 33425691 PMC7779734

[36] Vance DE Vance JE. Biochemistry of Lipids and Membranes. Menlo Park, CA: Benjamin/Cummings Publishing Company (1985). 593 p.

[37] Mohsen MK Gaafar HMA Khalafalla MM Yousif AM. Effect of rumen protected choline supplementation on digestibility, rumen activity and milk yield in lactating Friesian cows. Slovak J Anim Sci. (2011) 44:13–20. 10.1017/S2040470010001597

[38] Martínez-Aispuro JA Sánchez-Torres MT Figueroa-Velasco JL Cordero-Mora JL. Recommendation of choline inclusion in lambs' diet. Agro Productividad. (2021) 14:1951. 10.32854/agrop.v14i6.1951

[39] Dong L Jin Y Cui H Yu L Luo Y Wang S. Effects of diet supplementation with rumen-protected betaine on carcass characteristics and fat deposition in growing lambs. Meat Sci. (2020) 166:108154. 10.1016/j.meatsci.2020.108154 32330830

[40] Ueland PM. Choline and betaine in health and disease. J Inherit Metab Dis. (2011) 34:3–15. 10.1007/s10545-010-9088-4 20446114

[41] Morrissey PA Kerry JP. Lipid Oxidation and the Shelf-Life of Muscle Foods. In: Steele R, editor. Understanding and Measuring the Shelf-Life of Food. Cambridge: Woodhead Publishing Ltd (2004). p. 357–95. 10.1533/9781855739024.2.357

